# Mitochondria, Energetics, Epigenetics, and Cellular Responses to Stress

**DOI:** 10.1289/ehp.1408418

**Published:** 2014-08-15

**Authors:** Daniel T. Shaughnessy, Kimberly McAllister, Leroy Worth, Astrid C. Haugen, Joel N. Meyer, Frederick E. Domann, Bennett Van Houten, Raul Mostoslavsky, Scott J. Bultman, Andrea A. Baccarelli, Thomas J. Begley, Robert W. Sobol, Matthew D. Hirschey, Trey Ideker, Janine H. Santos, William C. Copeland, Raymond R. Tice, David M. Balshaw, Frederick L. Tyson

**Affiliations:** 1 Division of Extramural Research and Training, National Institute of Environmental Health Sciences (NIEHS), National Institutes of Health (NIH), Department of Health and Human Services (DHHS), Research Triangle Park, North Carolina, USA; 2 Nicholas School of the Environment, Duke University, Durham, North Carolina, USA; 3 Free Radical and Radiation Biology Program, Department of Radiation Oncology, Carver College of Medicine, University of Iowa, Iowa City, Iowa, USA; 4 University of Pittsburgh Cancer Institute, Hillman Cancer Center, University of Pittsburgh, Pittsburgh, Pennsylvania, USA; 5 Massachusetts General Hospital Cancer Center, Harvard Medical School, Boston, Massachusetts, USA; 6 Department of Genetics, University of North Carolina at Chapel Hill, Chapel Hill, North Carolina, USA; 7 Laboratory of Environmental Epigenetics, Exposure Epidemiology and Risk Program, Harvard School of Public Health, Boston, Massachusetts, USA; 8 SUNY College of Nanoscale Science and Engineering, Albany, New York, USA; 9 Department of Pharmacology and Chemical Biology, University of Pittsburgh School of Medicine, Pittsburgh, Pennsylvania, USA; 10 Duke University Medical Center, Durham, North Carolina, USA; 11 Department of Medicine, and; 12 Department of Bioengineering, University of California San Diego, La Jolla, California, USA; 13 Laboratory of Molecular Carcinogenesis, and; 14 Laboratory of Molecular Genetics, NIEHS, NIH, DHHS, Research Triangle Park, North Carolina, USA; 15 Biomolecular Screening Branch, Division of the National Toxicology Program, NIEHS, NIH, DHHS, Research Triangle Park, North Carolina, USA

## Abstract

Background: Cells respond to environmental stressors through several key pathways, including response to reactive oxygen species (ROS), nutrient and ATP sensing, DNA damage response (DDR), and epigenetic alterations. Mitochondria play a central role in these pathways not only through energetics and ATP production but also through metabolites generated in the tricarboxylic acid cycle, as well as mitochondria–nuclear signaling related to mitochondria morphology, biogenesis, fission/fusion, mitophagy, apoptosis, and epigenetic regulation.

Objectives: We investigated the concept of bidirectional interactions between mitochondria and cellular pathways in response to environmental stress with a focus on epigenetic regulation, and we examined DNA repair and DDR pathways as examples of biological processes that respond to exogenous insults through changes in homeostasis and altered mitochondrial function.

Methods: The National Institute of Environmental Health Sciences sponsored the Workshop on Mitochondria, Energetics, Epigenetics, Environment, and DNA Damage Response on 25–26 March 2013. Here, we summarize key points and ideas emerging from this meeting.

Discussion: A more comprehensive understanding of signaling mechanisms (cross-talk) between the mitochondria and nucleus is central to elucidating the integration of mitochondrial functions with other cellular response pathways in modulating the effects of environmental agents. Recent studies have highlighted the importance of mitochondrial functions in epigenetic regulation and DDR with environmental stress. Development and application of novel technologies, enhanced experimental models, and a systems-type research approach will help to discern how environmentally induced mitochondrial dysfunction affects key mechanistic pathways.

Conclusions: Understanding mitochondria–cell signaling will provide insight into individual responses to environmental hazards, improving prediction of hazard and susceptibility to environmental stressors.

Citation: Shaughnessy DT, McAllister K, Worth L, Haugen AC, Meyer JN, Domann FE, Van Houten B, Mostoslavsky R, Bultman SJ, Baccarelli AA, Begley TJ, Sobol RW, Hirschey MD, Ideker T, Santos JH, Copeland WC, Tice RR, Balshaw DM, Tyson FL. 2014. Mitochondria, energetics, epigenetics, and cellular responses to stress. Environ Health Perspect 122:1271–1278; http://dx.doi.org/10.1289/ehp.1408418

## Introduction

**Figure 1:**
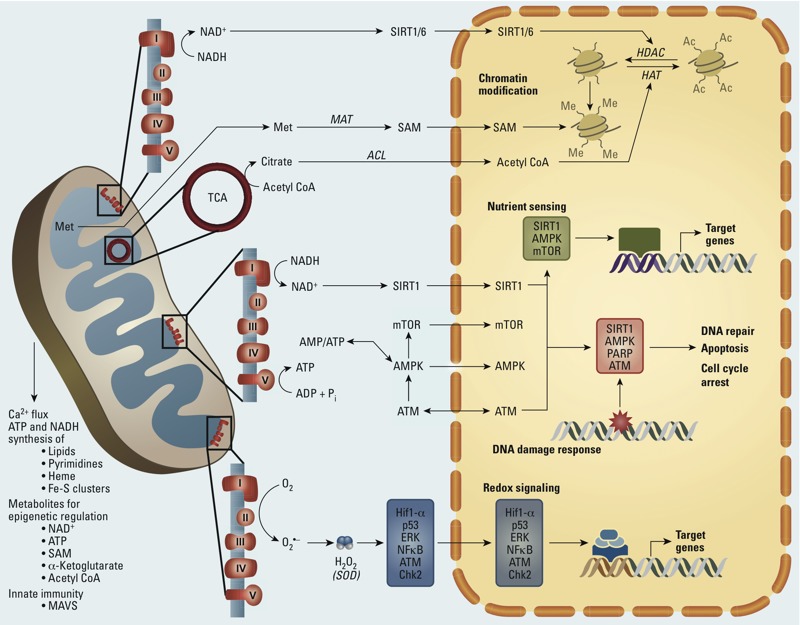
Mitochondria–nuclear signaling. Abbreviations: ACL, ATP citrate lyase; AMPK, AMP-activated protein kinase; Acetyl CoA, acetyl coenzyme A; ERK, extracellular signal-regulated kinase; HATs, histone acetyltransferases; HDACs, histone deacetylases; Hif1-α, hypoxia inducible factor 1 alpha; MAT, methionine adenosyltransferase; MAVS; mitochondrial antiviral signaling protein; Met, methionine; mTOR, mechanistic target of rapamycin; NFκB, nuclear factor kappa B; PARP, poly(ADP-ribose) polymerase 1; SAM, *S*‑adenosylmethionine; TCA, tricarboxylic acid cycle. Mitochondrial functions include cellular energy production via ATP generation, Ca^2+^ metabolism, synthesis of macromolecules, generation of metabolites for epigenetic regulation, and innate immune response to viral infection through MAVS. Nuclear–mitochondria signaling is mediated by numerous pathways, including epigenetic regulation/chromatin modification via sirtuins (e.g., SIRT1 and SIRT6), HDACs, and HATs, which require acetyl CoA from the TCA cycle; nutrient sensing through the AMPK and mTOR signaling pathways; DDR mediated by PARP, ATM, SIRT1, and AMPK; and redox signaling through overlapping pathways mediated by ATM/Chk2, p53, Hif1-α, ERK, and NFκB.

Mitochondria are critical to normal cell and organ function; they play a key role in metabolic homeostasis, in part, because of their central role in energy production. They also play major roles in apoptosis, control of cytosolic Ca^2+^ (calcium ion) levels, lipid homeostasis, steroid synthesis, generation of Fe-S (iron–sulfur) centers, heme synthesis, innate immune response, and metabolic cell signaling (Cheng and Ristow 2013; Papadopoulos and Miller 2012; Suen et al. 2008; Tait and Green 2012; Zemirli and Arnoult 2012) (Figure 1). Thus, it is not surprising that mitochondrial dysfunction underlies many diseases (e.g., Leber hereditary optic neuropathy, Alpers’ syndrome) that are individually rare but collectively occur at a rate of roughly 1 in 4,000 individuals. Mitochondrial dysfunction and altered organellar regulation are also associated with some more common diseases, including cancers, neurodegenerative diseases, and type 2 diabetes (Fariss et al. 2005; Van Houten et al. 2006).

Mitochondrial proteins and mitochondrial DNA (mtDNA) are vulnerable to damage by reactive oxygen species (ROS) because ROS are produced during normal energy production by oxidative phosphorylation and ATP generation via the electron transport chain (ETC). Mitochondria are also susceptible to insult from multiple natural and synthetic compounds that exert their toxicity by *a*) altering mtDNA integrity, *b*) inhibiting complexes in the ETC, *c*) modifying membrane potential, *d*) affecting Ca^2+^ transport, and *e*) activating proapoptotic signaling (Meyer et al. 2013). Furthermore, gene–environment interactions are critical in these events: Exposures to chemicals that are otherwise innocuous may cause disease and death in people with mutations or gene variants that affect mitochondrial function (Guan 2011; Silva et al. 2008). The association of mitochondrial dysfunction with numerous chronic diseases may reflect, in part, the vulnerability of mitochondria to environmental and exogenous insults. In support of the Tox21 high-throughput screening program [National Institutes of Health (NIH) 2014b], Attene-Ramos et al. (2014) used a cell-based assay to identify 1,222 compounds (~ 15% of the total compounds tested) that reduced mitochondrial membrane potential. However, whether such compounds act in a direct or indirect manner on mitochondrial functions—and what the specific mitochondrial targets are for these stressors—remains unclear. A systems approach, which enables real-time integration of the role of mitochondrial function in multiple cellular sensing and response pathways—including redox signaling, nutrient sensing, and multiple biosynthetic pathways—would enhance our understanding of exposure-induced mitochondrial dysfunction. Recent studies have illustrated the extent to which mitochondria are integrated into cellular responses under changing environments (Meyer et al. 2013).

In this review, we discuss the concept of cross-talk between mitochondria and other cellular pathways in response to environmental stress. A more comprehensive understanding of cellular stressors on acute responses and disease pathologies based on the role of energetics and other mitochondrial functions interacting with key pathways will be critical to elucidating their contribution to health outcomes.

## Methods

The National Institute of Environmental Health Sciences (NIEHS) sponsored the Workshop on Mitochondria, Energetics, Epigenetics, Environment, and DNA Damage Response on 25–26 March 2013. A major goal of the meeting was to discuss mitochondria–cell signaling in different cell types and organisms, with changing stress conditions, in order to understand the relationship between mitochondrial function, cellular homeostasis, and disease. A series of roundtable discussions resulted in a set of recommendations and research opportunities to promote this field of research. In this review, we further consider the state of the science discussed at the workshop.

## Discussion

**Figure 2:**
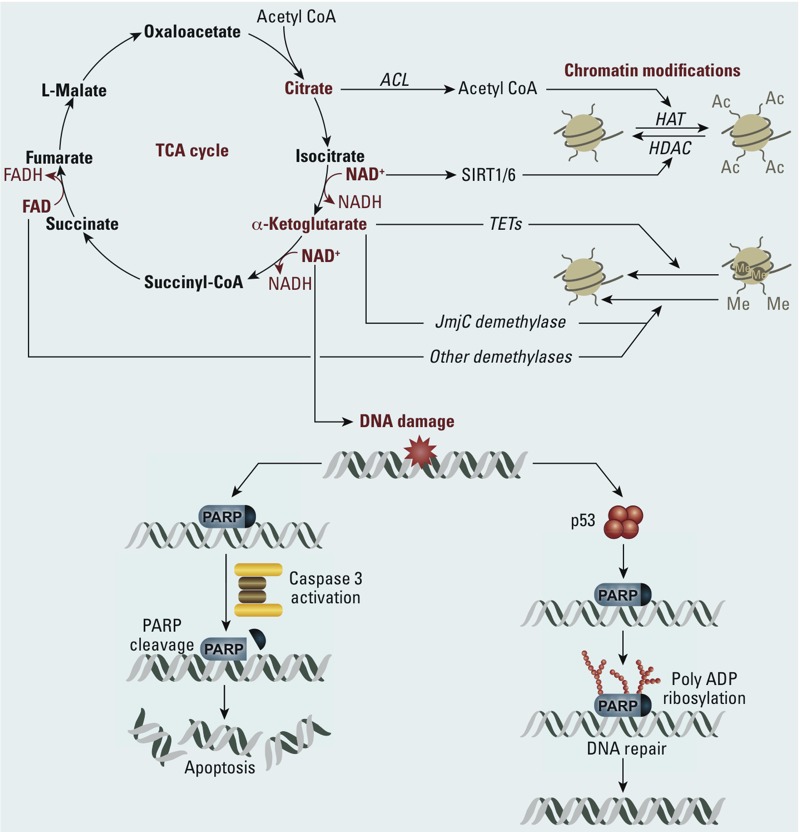
Tricarboxylic acid (TCA) cycle metabolites. Abbreviations: Acetyl CoA, acetyl coenzyme A; ACL, ATP citrate lyase; HAT, histone acetyltransferase; HDAC, histone deacetylase; PARP, poly(ADP-ribose) polymerase 1. Metabolites formed in the TCA cycle are important substrates for proteins involved in epigenetic regulation and DDR. Citrate, converted to Acetyl CoA by ATP citrate lyase (ACL) in the cytoplasm and nucleus, is required for histone acetylation by HATs. NAD^+^ is required for SIRT1 activity and PARP activation in DDR and apoptosis pathways, and α-ketoglutarate is a cofactor for the TET family of dioxygenases that convert 5-methylcytosine to 5-hydroxymethylcytosine, which can be replaced by unmethylated cytosine via DNA repair activities (deamination and BER).

*Mitochondrial function and epigenetics*. Mitochondria provide key metabolites [including but not limited to β-nicotinamide adenine dinucleotide (NAD^+^), ATP, α-ketoglutarate (α-KG; also called 2-oxoglutarate, 2-OG), and acetyl coenzyme A (acetyl CoA)] that are co-substrates required for numerous transcriptional and epigenetic processes (e.g., chromatin remodeling, histone modifications, nucleosome positioning) (Cyr and Domann 2011; Donohoe and Bultman 2012; Martinez-Pastor et al. 2013) (Figure 2). Although it is anticipated that mitochondria may play a critical role in regulating gene expression, data demonstrating that mitochondrial metabolites are rate limiting for epigenetic modifiers are still lacking (Cai et al. 2011; Lu and Thompson 2012). Nevertheless, increasing evidence points to the role of mitochondria in modulating the epigenome. For instance, neomorphic gain of function mutations in isocitrate dehydrogenase (*IDH1* or *IDH2*) results in the conversion of α-KG to 2-hydroxyglutarate, which can inhibit DNA demethylases and alter gene expression patterns (Schulze and Harris 2012). In cases of acute myeloid leukemia and glioblastoma, the *IDH1/2* mutation results in the formation of 2-OH-glutarate, which is a competitive inhibitor of α-KG–dependent processes, especially demethylation of histones (Turcan et al. 2012; Ward et al. 2012). Histone acetylation has multiple roles in transcriptional regulation, including the provision of binding sites for proteins containing bromodomains, alteration of chromatin subnuclear localization and structure, and neutralization of histone positive charges (Wellen et al. 2009). Ladurner (2009) pointed out that Wellen et al. (2009) showed how mitochondrially generated citrate can serve as a substrate for the production of nuclear acetyl Co-A. A few studies have demonstrated the requirement of a pool of acetyl Co-A for global histone acetylation by histone acetyltransferases (Cai et al. 2011; Takahashi et al. 2006). In addition to the direct provision of substrates, mitochondria can influence epigenetic signaling indirectly through ROS generation [e.g., hydrogen peroxide (H_2_O_2_)] (Desouki et al. 2005; Smiraglia et al. 2008). Epigenetic alterations in response to ROS may in turn result in altered expression of genes that regulate mitochondrial metabolism. In addition to endogenous metabolite levels, metals and other environmental pollutants have been shown to alter epigenetic patterns, including global DNA methylation and histone modifications *in vitro* and *in vivo* (Byun et al. 2013; Hou et al. 2012).

Metabolic epigenetics refers to nuclear alterations of chromatin and other factors that regulate gene expression resulting from changes in mitochondrial energetics and metabolism. The resulting metabolites, in turn, mediate gene expression changes that control cellular processes, including energy homeostasis (Wallace and Fan 2010). Thus, energy status and metabolism are able to modulate epigenetic programming via chromatin structural changes and dynamics, DNA methylation, histone modifications, and noncoding RNA expression. Epigenetic modifiers include DNA methyltransferases, histone acetyltransferases, histone deacetylases, sirtuins (SIRTs), histone lysine demethylases, poly(ADP-ribose) polymerases, and others that work coordinately to regulate gene expression. Multiple changes in cellular energetics and epigenetic processes that are mediated by factors including SIRTs and chromatin states (Figure 2) have been observed in investigations of complex diseases. For instance, reprogramming of energy metabolism has been identified as an emerging hallmark of cancer (Hanahan and Weinberg 2011; Nakajima and Van Houten 2013). Alterations that promote or enable a shift in mitochondrial metabolism toward aerobic glycolysis may predispose cells to a carcinogenic-type phenotype (Vander Heiden et al. 2009). For example, SIRT6 acts as a nutrient sensor by linking epigenetic gene silencing and cellular energetics in maintaining genome stability and tumor suppression. A recent study indicated that SIRT6 acts specifically in these processes as a corepressor of hypoxia-inducible factor 1-alpha (HIF1α) and MYC targets via both H3K9 and H3K56 deacetylation (Sebastian et al. 2012). This implicates SIRT6 as a tumor suppressor through its ability to down-regulate aerobic glycolysis in tumor cells (Ho et al. 2012). An emerging concept is that tumor cells are metabolically flexible and hypoxic regions of the tumor may display increased glycolysis and glucose utilization, whereas other well-vascularized regions of the tumor may show high levels of oxidative phosphorylation using different carbon sources (Ho et al. 2012; Nakajima and Van Houten 2013). For example, ovarian and prostate cancers show high levels of fatty acid beta-oxidation (Nieman et al. 2013).

Another central function of mitochondria is ROS signaling and sensing. Indeed, mitochondria operate as redox sensors that can alter energy states in response to the chemical environment of the cell and relative levels of endogenous metabolites such as iron(II), succinate, and ascorbate, as well as various forms of ROS. However, how ROS sensing is mediated by mitochondrial function and how different ROS sensing pathways overlap are not well understood. Certainly, changes in redox states can influence DNA methylation (Hitchler and Domann 2009) because the oxidation of 5-methylcytosine to 5-hydroxymethylcytosine in CpGs can perturb recognition by methyl-binding proteins and subsequently alter methylation patterns and epigenetic regulation (Hitchler and Domann 2012).

Dietary changes, including carbon sources, can also affect mitochondrial function and epigenetics (Zhang et al. 2012). Butyrate, for example, is a very short-chain fatty acid that has multiple roles in the cell and serves as a key energy metabolite, histone deacetylase inhibitor, and—via the generation of acetyl-CoA—as a histone acetyltransferase activator (Andriamihaja et al. 2009; Donohoe and Bultman 2012). Butyrate is generated by microbiota in the colon during the digestion of dietary fiber (Leschelle et al. 2000). Donohoe et al. (2011) demonstrated that in colon cells, the microbiota is a key regulator of energetics because normal colonocytes use bacterial butyrate as a primary aerobic energy substrate. Butyrate also inhibits cell proliferation in colon cancer cells and conversely stimulates growth in normal colon cells. These results suggest that normal cells and colon cancer cells utilize butyrate differently in a manner that affects epigenetic processes.

An additional epigenetic–mitochondrial interaction could be the alteration of mtDNA methylation by environmental stressors, although it is currently unclear whether mtDNA transcription is linked to altered mtDNA methylation in the same manner as is nuclear DNA (nDNA) methylation. Furthermore, effects of exposures on putative mitochondrial epigenetic states will generally occur in the context of direct effects on both mitochondrial and nuclear epigenetics; these effects may or may not be mechanistically linked. For example, what are the steps involved in response to air pollution? Is the mitochondrial response an early step in cellular reprogramming (e.g., an increase in mitochondrial content or biogenesis, followed by alterations in methylation of nuclear-encoded mitochondrial genes)? Recent human studies have demonstrated effects of air pollution exposure on mtDNA copy number, a marker that can be applied in large population studies and may reflect both mtDNA damage and dysfunction (Carugno et al. 2012; Hou et al. 2010, 2013; Janssen et al. 2012; Pavanello et al. 2013). There is growing evidence suggesting that air pollution exposure modifies methylation not only in the nDNA but also in the mtDNA (Baccarelli et al. 2009). Although this finding might help to identify individuals at higher risk of air pollution effects, including acute and long-term cardiorespiratory disease, lung cancer, and neurological effects, there are conflicting reports in the literature regarding the function of mtDNA methylation (Dzitoyeva et al. 2012; Hong et al. 2013; Iacobazzi et al. 2013). A fundamental question is whether cytosine methylation takes place in mtDNA, particularly in sequences that are rich in CpG dinucleotides. Intriguingly, recent evidence appears to suggest that methylation can occur in cytosines both in a CpG context and in cytosines that are not in CpG sites. Specifically, increased cytosine methylation has been observed in promoter regions of the mitochondria heavy strand located at the 5´-end of the D-loop (involved in DNA synthesis), suggesting a role in regulating mtDNA replication. Moreover, the observation of 5-hydroxymethylcytosine in mtDNA provides additional evidence that mtDNA may be epigenetically regulated. That this base has been established without the action of TET dioxygenases, which do not contain a mitochondrial targeting sequence, suggests other modes for demethylation and ultimately metabolic reprogramming that could be mediated via cross-talk with the nucleus (Bellizzi et al. 2013; Shock et al. 2011).

*Mitochondria and DNA damage response*. Mitochondrial functions are also tightly integrated with cellular responses to damage in both mtDNA and nDNA. Given the significant generation of ROS during normal mitochondrial functions, it is not surprising that base excision repair (BER), which repairs most oxidative DNA damage, is a critical DNA repair pathway in the maintenance of mtDNA integrity (Mandal et al. 2012; Maynard et al. 2010). Other DNA repair pathways that protect the nuclear genome—including mismatch repair as well as repair of DNA double-strand breaks through homologous recombination or nonhomologous end joining—may be active in mitochondria, but the specific roles for these pathways, or the proteins involved in maintaining mtDNA stability, are not clear (Alexeyev et al. 2013; Kazak et al. 2012). Nucleotide excision repair, which repairs damage resulting from many common environmental genotoxicants (Basu and Blair 2011) including polycyclic aromatic hydrocarbons, mycotoxins, and ultraviolet radiation, is not present in mitochondria (Kazak et al. 2012). Recent research is improving our understanding of the relationship between nDNA and mtDNA repair pathways, the effects of persistent mtDNA damage, and the energetic requirements for both nDNA and mtDNA DDR pathways.

Repair of oxidative and alkylation DNA damage in mitochondria through BER occurs in a manner similar to that of nDNA with several modifications: Gap filling in both short- and long-patch repair is carried out by polymerase gamma and its accessory subunits, DNA ligase III and EXOG (which carries out 5´ to 3´ exonuclease activity in long-patch BER) (Cymerman et al. 2008). Depletion of EXOG in human cell lines results in persistent single-strand breaks in mtDNA, increased mitochondrial dysfunction, and increased apoptosis (Tann et al. 2011). Similarly, DNA ligase III activity has been shown to be critical for mtDNA repair and cell survival (Simsek et al. 2011). In general, loss of BER activities, including EXOG or DNA ligase III, would be expected to cause single-strand breaks, leading to a decrease in mitochondrial transcription and subsequent defects in the ETC, and ROS production, ultimately leading to cell death or necrosis (Sharma et al. 2014). In support of this, intrinsic mtDNA repair defects are observed in the disease ataxia telangiectasia in which DNA ligase III levels are significantly reduced. This decrease in ligase III leads to slower kinetics of mtDNA repair, loss of mtDNA integrity, and ultimately mitochondrial dysfunction (Sharma et al. 2014).

Recent studies suggest that specific types of DNA damage have varying effects on mitochondrial function and cell survival. Furda (et al. 2012) demonstrated that given similar levels of mtDNA lesions in mouse embryonic fibroblasts, treatment with the alkylating agent methyl methanesulfonate had little effect on mitochondrial function, whereas H_2_O_2_-treated cells exhibited significant mtDNA loss, disruption of the ETC complex Vα subunit and complex 1 levels, and a decline in oxidative phosphorylation. Other studies have reported that DNA lesions generated from ultraviolet C radiation–treated *Caenorhabditis elegans* were not repaired but also did not persist indefinitely in mtDNA (Bess et al. 2012). The slow disappearance of these lesions was abrogated in nematodes in which expression of mitochondrial fusion, fission, and autophagy proteins was knocked down by RNAi (RNA interference) (Bess et al. 2012). Furthermore, these lesions resulted in mitochondrial dysfunction (Leung et al. 2013) that was exacerbated in the context of deficiencies in some mitochondrial fusion, fission, and autophagy proteins (Bess et al. 2012, 2013). Thus, other mitochondrial quality-control mechanisms, including fission, fusion, and mitophagy, are responsible for protecting mitochondrial function and tolerance of mtDNA lesions (Figge et al. 2012).

Increasingly, proteins typically thought of as mitochondrial have been found to have critical extra-mitochondrial “moonlighting” roles, and, conversely, proteins typically thought of as extra-mitochondrial have demonstrated mitochondrial effects. Qian et al. (2012) reported that inhibition of the mitochondria fission protein Drp1 causes cell cycle disruption, with G_2_ arrest, abnormal DNA content, aneuploidy, and other chromosome abnormalities in human cell lines. Effects on cell cycle progression were independent of mitochondrial energy metabolism and ROS generation. The underlying mechanism for Drp1 deficiency leading to G_2_/M arrest and aneuploidy is not yet known. However, it may be mediated by mitochondrial hyperfusion leading to aberrant cyclin E expression during G_2_ and replication stress that induces the G_2_/M checkpoint. Conversely, several studies have shown that the DDR protein ATM (ataxia-telangiectasia mutated) also functions in redox sensing, insulin signaling, and cellular energy balance through the *AMPK* (AMP-activated protein kinase) pathway (Ditch and Paull 2012), and appears to play an important role in mitochondrial homeostasis (Valentin-Vega and Kastan 2012). Thymocytes from *Atm*-null mice show altered mitochondrial morphology, elevated ROS levels, and decreased ETC activity and ATP production. Loss of *ATM* also leads to increased mitochondrial mass and oxygen consumption, suggesting impairment of mitophagy (Valentin-Vega et al. 2012). The recent observations that DNA ligase III levels are decreased in the absence of ATM may also explain these results (Sharma et al. 2014).

DDR pathways are highly energy dependent, with requirements for ATP and NAD^+^ during DNA damage sensing and repair activities. For example, poly(ADP-ribose) polymerase 1 (PARP1) plays a crucial role in multiple repair pathways, including BER, in sensing damage and initiating and completing repair of DNA lesions and DNA strand breaks. PARP1 activation requires NAD^+^ and serves to recruit repair activities to the damaged site. Incomplete repair (e.g., BER failure), possibly from PARP1 hyperactivation and cellular energy depletion, leads to cell death (Jelezcova et al. 2010; Tang et al. 2010). How cellular processes, including DDR, are regulated through PARP1 activation and alterations in NAD^+^ metabolites is not understood, but this is another example of critical interactions between mitochondrial function and energetics and cellular responses to stress.

Cells may also respond to extensive DNA damage through apoptosis, and mitochondria play a key role in this pathway through activation of BCL-2-associated X protein (BAX) or BCL-2 antagonist/killer (BAK) in response to proapoptotic signals including DNA damage. Activation of BAX and BAK leads to mitochondrial outer membrane permeabilization and release of cytochrome *c*, which binds and activates proapoptotic factors that include APAF1, caspase 3, and caspase 7 (Tait and Green 2010). Autophagy, which acts in cellular detoxification, energy production, and anabolic processes under conditions of cellular stress, is also regulated by mitochondria. For example, under nutrient starvation and low ATP conditions, AMPK phosphorylates a number of autophagy-related proteins including ULK1 and the mTORC1 regulators, TSC2 and RAPTOR (Tait and Green 2012).

## Recommendations and Research Opportunities

The Mitochondria, Energetics, Epigenetics, Environment, and DNA Damage Response workshop defined key gaps in research and understanding regarding cross-talk between the nucleus and mitochondria. Research exploring the signaling associated with the DDR, epigenetics, and mitochondrial dynamics and energetics forms a basis for exploring the cross-talk between these pathways in environmentally mediated disease. In addition, key recommendations were identified for resources, infrastructure, and technologies needed to move this field forward. In particular, there is a need to move toward *in vivo*, real-time measures of metabolites with increased resolution as key indicators for unraveling the cross-talk between the nucleus and mitochondria. Some of the major recommendations from this workshop are presented below.

*Metabolomics and flux technologies*. The relationship between mitochondrial dynamics and energy metabolism is still poorly understood, and predicted paths from metabolomics are underdeveloped. Identifying the role of small molecules in mediating the cross-talk is approachable using today’s metabolomics technologies, including improvements to metabolomics technologies and enhanced training supported through the NIH Common Fund Metabolomics Program (NIH 2014a). However, there is a need for focused development to enable further studies. One key area is in improved flux analysis, which allows for the investigation of biological reactions at steady state through monitoring stable isotope levels in both *in vitro* and *in vivo* studies (Basu and Blair 2011; Gravel et al. 2014; Maher et al. 2012; Sauer 2006; Zamboni 2011). This technology needs to be developed to the level of other “omics” technologies, particularly by coupling it with transcriptomics and epigenomics data integration.

Further advances in technology will also be necessary to apply these technologies in high-throughput screening efforts. Improving three-dimensional imaging and new methods in sequencing mtDNA will be particularly important in this regard. For measuring mitochondrial function in intact cells and isolated mitochondria, the Seahorse Flux analyzer (Seahorse Biosciences) has revolutionized mitochondrial studies in terms of enabling high-throughput measurement of mitochondrial metabolism (Kembro et al. 2013; Qian and Van Houten 2010). However, other studies are needed to link these end points with changes in the mitochondrial proteome or metabolite profiles. Affinity purification mass spectrometry is a technology that can detect the effects of exposure on protein interactions in human cell culture. Several studies have employed magnetic resonance spectroscopy to monitor changes in mitochondrial metabolism in human patients with Friedreich ataxia (Nachbauer et al. 2013) and in controlled studies of the effects of exercise on muscle oxidative capacity in healthy subjects (Layec et al. 2013). For high-throughput screening, the Tox21 program is currently using a mitochondria membrane potential assay to screen large numbers of environmental compounds and drugs for effects on mitochondrial function (Attene-Ramos et al. 2014). As such intensive screening efforts continue, there is increasing need for enhanced support for infrastructure to allow storage of chemical-response data from high-throughput screening efforts, as well as from other assays, to be appropriately cataloged and published as a public resource.

Another fundamental need is the ability to precisely track free radicals and distinguish different types of ROS via their source and mode of generation in the cell. Oxidative damage is 5–10 times higher in mtDNA than in nDNA, and mitochondria are directly exposed to endogenous ROS. Yet it is not known how much mitochondrially generated H_2_O_2_ reaches the nucleus. The development of fluorescent probes will enable studies that will more accurately measure localized ROS and how different perturbations affect ROS. This will require collaborative, multidisciplinary expertise between chemists and cellular and molecular biologists. Success in applying these probes will also require the evolution of technologies for the imaging of metabolites in cells to enable studies of the subcellular localization of signaling activities. Having such tools to track small molecule and free radical diffusion will enable studies that can better address low-dose toxicant exposures that are relevant to disease pathogenesis.

*Human populations systems and experimental models*. The Mitochondria, Energetics, Epigenetics, Environment, and DNA Damage Response workshop highlighted a variety of resources available in human population studies and cell systems that might be particularly useful for understanding cross-talk between the mitochondria and nucleus, and between diverse biological pathways. Research involving childhood cancer survivors, many of whom show adverse health outcomes later in life (Hudson et al. 2013); progeria patients; and HIV patients treated with nucleoside analogs including AZT (azidothymidine) might offer opportunities to study the roles for altered mitochondrial function and energetics on other cellular pathways because many chemotherapeutics and nucleoside analogs cause mitochondrial damage (Cossarizza and Moyle 2004; Poirier et al. 2003). *In vitro* assays using human differentiated induced pluripotent stem (iPS) cells from patients with inherited mitochondrial defects and unaffected individuals could be used to evaluate differential sensitivity to mitochondrial toxicants and better understand tissue and cell specificity and tissue-specific thresholds for mitochondrial functions involved in highly heterogeneous mtDNA diseases (Fujikura et al. 2012; Hamalainen et al. 2013). In particular, reprogramming somatic cells from patients with mtDNA disorders can generate pluripotent stem cells with varying degrees of heteroplasmy and allows the creation of patient-specific pluripotent cells that retain the functional characteristics of donor cells, including disease-associated mtDNA (Cherry et al. 2013). Studying repair capacity for mitochondrial genetic variants in human populations is also helpful for understanding the genetic susceptibility underlying environmental exposures in diverse health outcomes resulting from mitochondrial dysfunction.

In some cases, research using model organisms has distinct advantages compared with human cell culture systems or other human population-based approaches. For example, yeast is an ideal model organism for understanding some human mitochondrial myopathies because of the advantages of monitoring fermentative growth in the case of respiratory-deficient mutants. Another strength of the yeast model is the ability to introduce multiple homoplasmic mitochondrial mutations for studying diseases such as Leber hereditary optic neuropathy, where multiple mitochondrial mutations are responsible for the pathologies (Meunier et al. 2013). *Drosophila melanogaster* has been useful in understanding the dysfunction of mitochondrial dynamics (especially mitochondria-shaping proteins) and its role in disrupting mitochondrial bioenergetics, which is implicated in neurodegenerative diseases (Debattisti and Scorrano 2013). Zebrafish models have been used to examine complex I and II deficiencies in both primary mitochondrial diseases, such as Charcot-Marie-Tooth, and in other neurodegenerative diseases associated with complex I or II deficiencies, such as Parkinson’s disease and Huntington’s disease. In addition, the zebrafish has emerged as a significant model for understanding the bioenergetics of environmentally relevant aquatic pollutants and in applications related to *in vivo* toxicity screening of chemicals affecting mitochondrial function (Bourdineaud et al. 2013; Pinho et al. 2013). The development of powerful new approaches in population-based mouse resources will also contribute to a greater understanding of the role of susceptibility and resistance to chemically induced mitochondrial dysfunction related to human disease (Flint and Eskin 2012). *In vitro* assays using embryonic stem/iPS cells from the Collaborative Cross/Diversity Outbred mouse models could identify genetic factors in differential sensitivity to toxicants, which can then be followed by *in vivo* studies to demonstrate functional relevance. Although cellular and genetic developmental processes associated with many mitochondrial functions are highly conserved between these model organisms and humans, cross-species extrapolation should focus on conserved pathways rather than on disease phenotypes to ensure that valid conclusions are drawn.

*Systems integration and focused investigations on cross-talk in environmental health*. As discussed above, cross-talk between the nucleus and mitochondria occurs partly via epigenetic pathways with many potential mitochondrial/epigenetic interactions, such as nDNA methylation effects on transcription of mRNA for mitochondrial proteins or the effects of mtDNA depletion on altered nDNA methylation. However, the many ways in which mitochondrial damage and dysfunction may be related to mitochondrial epigenetics and environmental disease are still poorly understood.

Additional research is needed in several important areas. Although nuclear CpG methylation receives substantial attention, methylation of mtDNA and its functional consequences are less well known. Unlike nDNA, mtDNA CpG sites are abundant, and a link between cytosine methylation and transcriptional alterations has not been established. Nevertheless, mtDNA methylation may represent an environmental target, with some mitochondrial toxicants potentially affecting cytosine methylation or mtDNA alkylation in general. Some evidence also suggests that mitochondrial DNA copy number may be an important environmental biosensor. Therefore, we need to understand the relevance of mtDNA copy numbers and mtDNA methylation to exposure-related human disease and whether relationships may exist with mtDNA haplogroups. There is some evidence that haplotypes of the mitochondrial genome affect stem cell differentiation and expression of genes involved in pluripotency, differentiation, and mitochondrial energy metabolism (Wittkopp et al. 2013). Protein acetylation is also an important regulatory mechanism, and diseases associated with mitochondrial dysfunction may also be related to protein acetylation, including type 2 diabetes, obesity, and cancer (Finkel et al. 2009; Hirschey et al. 2011; Sebastian et al. 2012). Protein acetylation is also common in the mitochondria, possibly affecting two-thirds of mitochondrial proteins, including many involved in energy-producing pathways, but the impact of such events are poorly understood. Clearly these data indicate a rich area for discovery.

A better understanding of how critical windows of susceptibility and developmental timing affect nucleus/mitochondria cross-talk is also needed. In general, the abundance of mtDNA in cells can be protective against damage, but this varies by cell type and developmental stage, which creates possible windows of vulnerability. Early developmental stages typically have lower mtDNA copy number, a phenomenon especially well-documented in the context of primordial germ cells (Carling et al. 2011; Jansen and de Boer 1998; Shoubridge and Wai 2007). Furthermore, periods of global demethylation during developmental windows may be particularly sensitive periods for effects of environmental stressors. Mapping the mitochondrial proteome, post-translational modifications (including phosphorylation, acetylation, sumoylation, parylation) in different cell types and stages of development, and combining mitochondrial proteome analysis with imaging will be informative, especially given the wide variability in mitochondrial form and function in different tissues and developmental stages (Johnson et al. 2007; Vafai and Mootha 2012). A better understanding of the reprogramming of mitochondrial genes during development might be gained through studying iPS cells and differentiated cells of interest. Participants of the Mitochondria, Energetics, Epigenetics, Environment, and DNA Damage Response workshop also emphasized the need for longitudinal prospective studies linking past exposures to mtDNA markers and phenotypes to better understand windows of susceptibility.

Recent studies have highlighted the overlap between DDR processes in the mitochondria and nucleus. In general, the presence of fewer DNA repair pathways in mitochondria confers greater vulnerability to damage, but we need to better understand DNA damage from environmental exposures in terms of effects on mitochondria versus only the nucleus. At present, it is difficult to determine when an exposure is a primary mitochondrial response (or a mitochondrial toxicant) rather than a mitochondrial response that occurs secondarily after a toxicant affects another subcellular target. There may be patterns of “omics” data (e.g., gene expression, genetic or protein interactions) that are indicative of distinct DNA repair mechanisms and could thus serve as a biomarker for particular types of DNA damage. In this regard, differential genetic networks are a powerful new tool for mapping the altered structure, as well as the function, of biological networks in response to environmental stresses (Ideker and Krogan 2012). In yeast, large networks of genetic interactions have been shown to be substantially rewired by different types (Guenole et al. 2013) and levels of DNA damage (Srivas et al. 2013), suggesting that the interaction pattern itself is a sensitive measure of how DNA damage is being handled and by what subpathways. It is likely that further insight into the cross-talk and signaling mechanisms between the mitochondria and the nucleus, as well as the interplay between mitochondria and toxicants, will warrant such a systems biology approach. Cross-disciplinary efforts between system biologists, biochemists, and basic molecular biologists will be needed to develop the tools and approaches needed to detect alterations in mitochondrial-cellular signaling under changing stress conditions. In addition, better systems analysis tools are needed, such as the Gene Set Enrichment Analysis tools developed by the Broad Institute (Mootha et al. 2003; Subramanian et al. 2005).

## Conclusions

Development and application of novel technologies, including new reagents for tracking the production and distribution of specific ROS, expanded fluxomics analysis, new proteomics and metabolomic approaches, and application of tools for studying DNA methylation and chromatin remodeling, will enable systems-based approaches to investigate how environmentally induced mitochondrial dysfunction affects other key pathways, including epigenetic regulation and DDR, and conversely, how alterations in these pathways affect mitochondrial function. A more comprehensive understanding of the cross-talk between mitochondria and other cellular response pathways will significantly improve our understanding of how cells sense and respond to environmental stress and will help to form a more solid basis for developing early biomarkers of environmentally related diseases.
